# Small Hsps as Therapeutic Targets of Cystic Fibrosis Transmembrane Conductance Regulator Protein

**DOI:** 10.3390/ijms22084252

**Published:** 2021-04-20

**Authors:** Stéphanie Simon, Abdel Aissat, Fanny Degrugillier, Benjamin Simonneau, Pascale Fanen, André-Patrick Arrigo

**Affiliations:** 1 INSERM, IMRB, Paris Est Creteil University, F-94010 Creteil, France; abdel.aissat@inserm.fr (A.A.); fanny.degrugillier@gmail.com (F.D.); benjamin.simonneau@inserm.fr (B.S.); pascale.fanen@inserm.fr (P.F.); 2 Département de Génétique, AP-HP, Henri Mondor Hospital, F-94010 Creteil, France; 3 Apoptosis, Cancer and Development Laboratory, Lyon Cancer Research Center, INSERM U1052-CNRS UMR5286, Claude Bernard University Lyon 1, Centre Léon Bérard, F-69008 Lyon, France; parrigo@mac.com

**Keywords:** cystic fibrosis, CFTR, sHsps, HspB1, HspB4, HspB5

## Abstract

Human small heat shock proteins are molecular chaperones that regulate fundamental cellular processes in normal and pathological cells. Here, we have reviewed the role played by HspB1, HspB4 and HspB5 in the context of Cystic Fibrosis (CF), a severe monogenic autosomal recessive disease linked to mutations in Cystic Fibrosis Transmembrane conductance Regulator protein (CFTR) some of which trigger its misfolding and rapid degradation, particularly the most frequent one, F508del-CFTR. While HspB1 and HspB4 favor the degradation of CFTR mutants, HspB5 and particularly one of its phosphorylated forms positively enhance the transport at the plasma membrane, stability and function of the CFTR mutant. Moreover, HspB5 molecules stimulate the cellular efficiency of currently used CF therapeutic molecules. Different strategies are suggested to modulate the level of expression or the activity of these small heat shock proteins in view of potential in vivo therapeutic approaches. We then conclude with other small heat shock proteins that should be tested or further studied to improve our knowledge of CFTR processing.

## 1. Introduction

Cystic fibrosis (CF) is one of the most common severe autosomal recessive genetic diseases in individuals of European descent. It predominantly affects the lungs and digestive system and is caused by more than 2000 mutations (www.genet.sickkids.on.ca, accessed on 1 February 2021) in the gene encoding the Cystic Fibrosis Transmembrane conductance Regulator (CFTR) protein, which regulates the hydroelectrolytic transport of exocrine, serous and mucus-secreting glands. As a consequence of these mutations, CFTR can be impaired in its functional activity, be absent or present in only a reduced quantity.

CFTR is a 168 kDa transmembrane protein (1480 amino acids) that belongs to the family of ATP-Binding Cassette transporters (ABC) [1]. These transporters share two hydrophobic transmembrane domains (TMD1 and TMD2) that constitute the pore of the channel, as well as two hydrophilic cytoplasmic ATP binding domains (NBD1 and NBD2) [2]. Among the different ABC transporters, the CFTR protein has a unique regulatory cytoplasmic domain modulated by protein kinases A and C [3]. Upon cAMP level increase, this R domain becomes phosphorylated by protein kinase A, hence allowing ATP to bind NBD1 and NBD2 domains which, through their dimerization, induce conformational changes in the transmembrane domains that result in the opening of the channel. The phenomenon is reversible since, upon ATP hydrolysis, the dissociation of NBD1-NBD2 dimers triggers the closure of the channel [4].

CFTR protein is mainly expressed at the apical membrane of epithelial cells lining the pancreas, lungs, sweat glands, intestine, liver and genital organs [5], where its major activity is to allow different anions to cross the plasma membrane, such as chloride (Cl^−^), bicarbonate (HCO_3_^−^) and iodide (I^−^) [6]. CFTR can also indirectly modulate the activity of other ionic channels, such as ENaC (Epithelial Na^+^ Channel) involved in the absorption of Na^+^ and subsequently water by epithelial cells [7]. It also plays a role in the activation and conductance of chloride channels (Outwardly Rectifying Chloride Channel, ORCC) and in reducing the activity the calcium-dependent chloride channels (Calcium-activated Chloride Channel, CaCC) [8,9]. Moreover, in the kidney epithelium, it regulates ROMK (Renal Outer Medullary Potassium) channels involved in the secretion of potassium (K^+^) and reabsorption of NaCl and water. Of particular interest, other studies reported that CFTR has an anti-inflammatory activity that can inhibit the transcriptional activity of NF-κB and subsequent IL-8 inflammatory cascade [10].

## 2. CFTR Biogenesis

CFTR is synthesized and inserted into the endoplasmic reticulum (ER) membrane in the form of a non-glycosylated precursor though CFTR mRNA translation by ER ribosomes. Folding of CFTR domains occurs independently and results in complex co-translation mechanisms and post-translational events. The first domain synthesized, which contains TM1 and TM2 segments, is TMD1. The TM2 segment is first recognized by the SRP signal recognition particle and inserted into the ER membrane. The Extra Cellular Loop 1 (ECL1) is then created by insertion of the TM1 segment. Thereafter, the other segments also work in pairs, such as TM3 with TM4 and TM5 with TM6, hence creating, respectively, the ECL2 and ECL3 peptide loops before their insertion into the ER membrane [11]. The NBD1 and R domains are then synthesized and folded followed by the TMD2 domain. As for TMD1, its insertion in the membrane occurs sequentially in pairs: segments TM7 with TM8, TM9 with TM10 and finally TM11 with TM12, hence creating ECL4, ECL5 and ECL6 loops, respectively [12]. NBD2 domain is the last one to be synthesized and folded [13,14].

**Figure 1:**
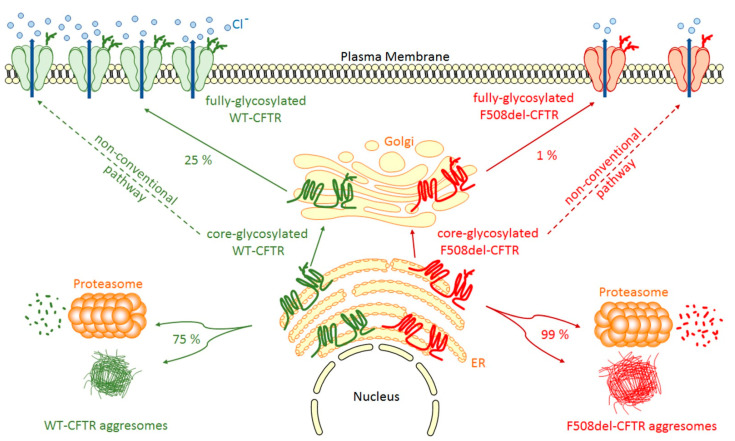
**WT- and F508del-CFTR biogenesis.** After synthesis in the Endoplasmic Reticulum (ER), the main part of core-glycosylated CFTR can form aggresomes or be recognized by the Endoplasmic Reticulum Quality Control (ERQC) system and directed to the proteasome to be degraded. CFTR escaped from ERQC undergoes different steps of glycosylation in the Golgi before being transported to the plasma membrane where it regulates ion channels. F508del-CFTR at the plasma membrane is less abundant and has only a low activity compared to WT-CFTR. A non-conventional trafficking of the CFTR protein can be activated when the conventional trafficking is blocked allowing expression of core-glycosylated CFTR at the plasma membrane.

Once inserted in the ER membrane, CFTR undergoes different steps of glycosylation and maturation (Figure 1) that are under the control of the Endoplasmic Reticulum Quality Control (ERQC) system where chaperone proteins like Hsp70, Hsc70, Hsp90, calnexin, Hdj-1 and Hdj-2 play major roles [15,16,17,18]. For example, the system can traffic CFTR to the Golgi by COPII vesicles [19] or trigger CFTR to the proteasome degradation pathway in cases of improper folding. This pathway depends on the Endoplasmic Reticulum-Associated Degradation (ERAD) system triggered by Hsp70 and Hsc70 [18]. From cis-, medial-, to trans-Golgi by COPI vesicles, CFTR continues to mature through post-translational modifications and specific glycosylation steps. During its maturation, three forms of CFTR are synthesized: the non-glycosylated A form (130 kDa), the core-glycosylated B form (150 kDa), which corresponds to the immature form and the fully-glycosylated C form (170 kDa), which is the mature form of the CFTR protein transported by vesicles to the plasma membrane. However, a major drawback is that CFTR biogenesis is not an efficient process with only about 25% of the synthesized proteins that are fully matured and active at the apical plasma membrane of epithelial cells. Hence, about 75% of the neosynthesized CFTR does not reach the final stage of maturation and is degraded by the ERAD coupled proteasome ubiquitin pathway (Figure 1). This peculiar property of wild-type CFTR is a direct consequence of its poor folding efficiency, a characteristic which is aggravated by mutations in its coding sequence. For example, among the many deleterious CFTR mutations, the deletion of phenylalanine at position 508 induces 99% (instead of 75% for wild-type protein) of F508del-CFTR variant to be destroyed before maturation (Figure 1) [20,21]. In 1998, a key experiment clearly demonstrated the weak folding ability of CFTR [22]. It was found that impairment of proteasome activity led to the accumulation of stable, high molecular weight, detergent-insoluble, multi-ubiquitinated forms of CFTR that were termed, for the first time, aggresomes. This term refers to an aggregation of misfolded proteins when the degradation system is overwhelmed, a prominent pathological feature also observed in neurodegenerative diseases. Additionally, a non-conventional trafficking of the CFTR protein can be activated when the conventional trafficking is blocked. As an example, ER stress has been shown to induce cell surface trafficking of the wild-type and F508-CFTR B forms via a Golgi ReAssembly Stacking Proteins (GRASP)-dependent pathway [23,24,25]. The detailed identification of proteins playing key roles in the processing of CFTR is of importance since they can be novel therapeutic targets [26].

## 3. Heat Shock Proteins Are Involved in the Quality Control of CFTR

In response to stresses that alter protein conformation, cells figure back by expressing chaperone proteins that make them return to a state of homeostasis. This cell response, which exists in both prokaryotes and eukaryotes, is characterized by the synthesis of heat shock proteins (Hsps), also called stress proteins. The main function of the different Hsps is to interact and chaperone immature proteins that in normal life or following stress or specific mutations, do not reach their final three-dimensional structure and help them to find a correct conformation. Moreover, through their chaperone activity, Hsps can stimulate their maturation, stability and transport and, in the case of irreversibly aggregated proteins, their degradation by the ubiquitin-proteasome machinery or autophagy. Of major importance, in physiological conditions, some Hsps are constitutively expressed and can therefore protect against apoptotic cell death and oxidative injuries, maintain and protect cytoskeleton integrity and play roles in differentiation, cell cycle, signal transduction, development, aging and tumorigenesis. In these different processes, each Hsp has a specific role which is often carried out in coordination with other chaperones.

Hsps are classified in families: the high molecular weights ones (Hsp110, Hsp105, Hsp90, Hsp70 and Hsp40) which bear ATP-dependent chaperone activities and small Hsps (sHsps) characterized by molecular weights in the 12 to 43 kDa range and their α-crystallin domain in their C-terminal region [27]. The human family of sHsps contains ten members (HspB1 to HspB10) that are constitutively expressed in specific tissues with variable levels of expression [28,29,30]. Some of them (HspB1, HspB5 and HspB8) show a stimulated expression in response to stress that alter protein conformation, such as heat shock, and are therefore considered true heat shock proteins. Concerning their constitutive expression, sHsps can be separated into two groups: those that have an almost ubiquitous expression (HspB1, HspB5, HspB6 and HspB8) and those that exhibit a more tissue-specific expression (HspB2, HspB3, HspB4, HspB7, HspB9 and HspB10). Some of them bear a chaperone activity (HspB1, HspB4, HspB5, HspB6, HspB7 and HspB8) which is different from that of the high molecular weight Hsps since it is ATP-independent. Through their chaperone activity and ability to interact with specific client polypeptides these sHsps can modify their activity and/or half-life [31,32].

Numerous studies have revealed that a large number of cytoplasmic and ER luminal chaperones, and among them many constitutively expressed Hsps, are essential for CFTR biogenesis. For example, some of the high molecular weight Hsps, like Hsp90, Hsp40 and the Hsp70 nucleotide exchange factor (NEF) HspBP1, are involved in the folding of CFTR through a mechanism called “the Hsp70-dependent folding of CFTR” [15,33,34,35,36,37,38,39,40]. It is also interesting to note that the inhibition of Hsp40 expression increases the level of F508del-CFTR mutant, suggesting, and in contrast to Hsp70 and Hsp90, a negative regulation of CFTR folding by this chaperone [41]. As such, it is not surprising that strategies to mimic or modulate Hsps in the context of CF have rapidly emerged. Thus, as early as 1998, the first clinical trial of F508del-CFTR homozygous patients was conducted with the administration of the chemical chaperone 4-phenylbutyrate (4-PBA, CAS number: 1716-12-7) [42]. This trial was performed following encouraging results showing that, in vitro, 4-PBA induces the recovery CFTR channel function at the plasma membrane of F508del-CFTR airway epithelial cells [43]. Unfortunately, the weak positive effect observed on chloride transport by nasal potential difference (NPD) of the patients was not associated with significant reduction of sweat chloride concentrations. It was later established that 4-PBA has a complex chaperone mechanism of action that can, for example, down-regulate constitutive Hsc70 and up-regulate inducible Hsp70 via the transcription factor STAT-3 (signal transducer and activator of transcription-3) [35,44]. These data illustrate the complexity of identifying molecules with a specific action on a given Hsp without impacting the other Hsps. Moreover, Hsps are very versatile chaperones whose effects can be reversed from one cell type, tissue or pathology to another. For example, in Idiopathic Pulmonary Fibrosis (IPF), inhibition of Hsp90 is a promising strategy at the late stage of the disease [45], whereas in CF benzoquinone ansamysin molecules that inhibit this Hsp block the maturation of nascent CFTR and strongly accelerate its degradation by the proteasome [34], a phenomenon which depends on Hsp90 co-chaperone partner Aha I (Activator of Hsp90 ATPase activity). This interesting observation extends the possibilities to pharmacologically inhibit Hsp90-mediated proteolysis of F508del-CFTR and rescue its activity. In that respect, Ihrig et al. have isolated two Hsp90-Aha1 inhibiting drugs that restore chloride channel activity in cells expressing the mutant F508del-CFTR protein [46]. Moreover, their effects were most effective in combination with the corrector Lumacaftor (VX-809), thus opening new roads to develop drugs to treat cystic fibrosis patients. Hence, a better understanding of the specific roles of each Hsp at the different steps of CFTR biogenesis can lead to opportunities aimed at therapeutically stimulating the weak folding ability of this protein.

Here, we have reviewed the roles played by three chaperones, the small heat shock proteins HspB4, HspB1 and HspB5, in the biogenesis, maturation and activity of CFTR as well as towards some of its mutated forms, the aim being to evaluate the therapeutic potential of compounds or conditions that modulate the activity of these chaperone proteins.

## 4. Small Hsps as Modulators of CFTR

### 4.1. HspB4 (αA-Crystallin)

**Figure 2:**
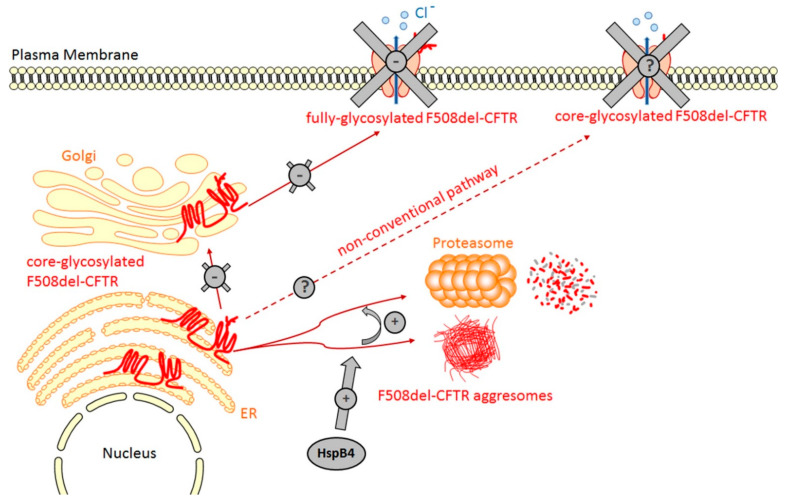
**HspB4 promotes the degradation of F508del-CFTR.** The transient expression of HspB4 reveals that it is able to bind F508del-CFTR. In vitro experiments have revealed that, through the binding of the NDB1 domain, HspB4 probably maintains F508del-CFTR in a soluble form that facilitates its degradation by the proteasome.

The HspB4 protein is a major protein of the mammalian lens where it associates with HspB5 (αB-crystallin) to form the α-crystallin dimer essential for lens transparency [47]. HspB4 is also weakly expressed in the pancreas where it negatively regulates tumorigenesis [48]. Mutations in HspB4 gene lead to peripheral and nuclear cataract diseases [49,50,51,52]. Historically, HspB4 was the first sHsps to have been studied in the CF context [53]. In 2007, in a study on the degradation of wild-type and mutant CFTR by the ERAD system, Ahner et al. demonstrated (in HEK and Human Embryonic Kidney cells devoid of HspB4 constitutive expression) that the transient expression of HspB4 increased the degradation of the F508del-CFTR mutant but not the wild-type protein. This intriguing observation suggests that HspB4 can distinguish wild-type CFTR from its mutated form. In addition, HspB4, that preferentially interacts and co-precipitates with F508del-CFTR, decreases the aggregation of the NBD1 domain and therefore maintains the solubility of F508del-CFTR during its associated degradation by the ERAD machinery. Finally, it was proposed that HspB4 recognizes F508del-CFTR defects only when this polypeptide is completely synthesized (Figure 2) [53].

Of interest, the role of HspB4 towards ER synthesized ions channels may not be restricted to the ERAD of CFTR since, in mouse collecting duck cells, this chaperone can recognize epithelial Na^+^ channels (ENaC) subunits at ER quality control checkpoints and target ENaC subunits for ER-associated degradation [54].

### 4.2. HspB1 (Hsp27)

Mammalian HspB1, also called Hsp27, which was for decades the most investigated small Hsp, has been extensively studied in the context of CF. Various basal levels of HspB1 constitutive expression have been observed in most human tissues and cellular types; an expression particularly intense in several cancer cells types [55,56,57,58]. Moreover, since it is a true heat shock protein, its level of expression is greatly stimulated in response to stress that impair protein conformation [59,60]. In stress conditions, HspB1, which is regulated by complex oligomerization and phosphorylation events, binds and stores misfolded proteins to avoid their aggregation before they can regain their correct folding through the action of ATP-dependent chaperones, such as Hsp70 or Hsp90. In cases of irreversibly impaired polypeptides, HspB1, together with the other chaperones, can promote their ubiquitination and proteasomal degradation machinery [60,61]. In unstressed cells, different oligomeric and phosphorylated forms of HspB1 [62,63] interact with numerous different client proteins in order to modulate their maturation, activity or half-life [31,64,65]. In humans, HspB1 mutants can cause neuronal pathologies, such as distal hereditary motor neuropathy and hereditary peripheral neuropathy, also known as Charcot–Marie–Tooth disease type 2F [66,67].

**Figure 3:**
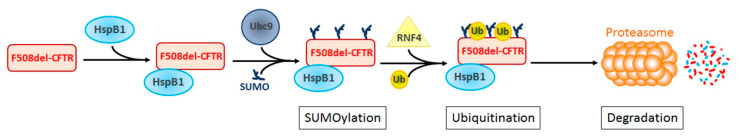
**HspB1 promotes the degradation by the proteasome of F508del-CFTR through SUMO and ubiquitin pathways.** HspB1 interacts specifically with the NDB1 domain of F508del-CFTR. This interaction favors the recruitment of UBC9 and the subsequent SUMOylation of F508del-CFTR. SUMOylated F508del-CFTR-HspB1 complex is recognized by the ubiquitin ligase RNF4, known to ubiquitinate SUMOylated proteins, thus causing their degradation by the proteasome.

In 2006, a proteomic analysis of nasal cells from healthy and F508del-CFTR patients revealed changes in the level of expression of 65 proteins. Among the most affected ones, HspB1 was surprisingly twice as low in expression in the F508del mutant cells compared to healthy cells [68]. At that time, it was hypothesized that the level of HspB1 expression could be linked to the level of functional CFTR. This observation prompted further studies dealing with the role of HspB1 toward CFTR mutants. Studies performed by Ahner et al. [69,70] thus identified HspB1 as being involved in a post-translational modification of F508del-CFTR, that is its SUMOylation which promotes its degradation (Figure 3). It was shown that HspB1 binds the NBD1 domain of F508del-CFTR and interacts with the SUMO-E2 enzyme, UBC9. This protein complex promotes the SUMOylation of mutant CFTR via the recruitment of SUMO-2 and / or SUMO-3. The resulting poly-SUMO chains are then recognized by the ubiquitin ligase RNF4, known to ubiquitinate SUMOylated proteins, thus causing their degradation by the proteasome [69,70].

In addition, these studies revealed that inhibition of HspB1 expression or F508del-CFTR SUMOylation by overexpressing Senp1 (Sentrin-specific protease 1), a polypeptide that specifically catalyzes SUMO maturation, stimulated the accumulation of CFTR mutants but had no effect towards wild-type CFTR [69,70,71,72,73].

### 4.3. HspB5 (αB-Crystallin)

In addition to being part, with HspB4, of the lens α-crystallin dimer, HspB5 constitutive expression is almost ubiquitous, including in lung tissue. As HspB1, this protein interacts with specific protein clients and modifies their activities [31,32]. The aim of the recent studies performed in our laboratory was to analyze the behavior of HspB5 towards CFTR mutants [74]. Could it, as HspB1 and HspB4, amplify the degradation of the F508del-CFTR mutant, have no effect or improve the maturation, transport and/or stability of F508del-CFTR? This last assumption is based on a study published in 2013 which revealed that HspB5 was able to rescue the folding and compartmentalization of two misfolded transmembrane proteins (Frizzled4-Fz4-FEVR mutant responsible for an autosomal dominant form of familial exudative vitreoretinopathy and ATP7B-H1069Q copper transporter mutant associated with a common form of Wilson’s disease) [75].

**Figure 4:**
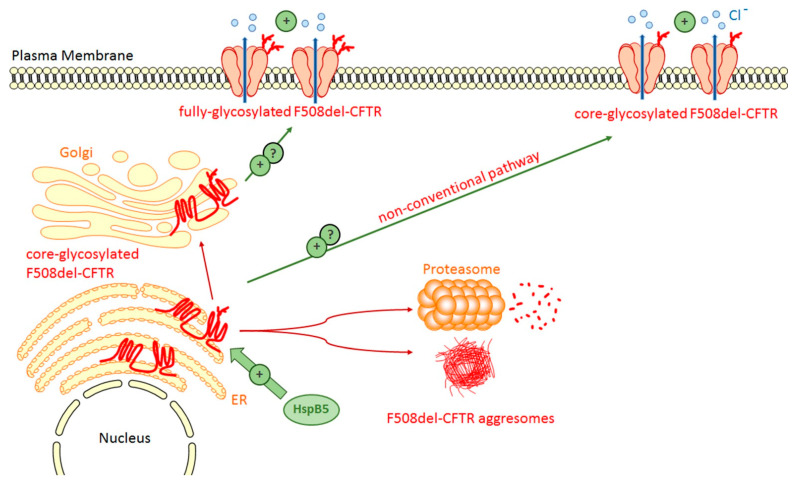
**HspB5 favors the rescue of F508del-CFTR.** The transient expression of HspB5 increases the stability of core-glycosylated F508del-CFTR thus enhancing the level of F508del-CFTR at the plasma membrane and its activity. The type of glycosylated F508del-CFTR form at the plasma membrane is currently not determined. One phosphomimic mutant of HspB5 (S19D, S45A, S59D) is more effective than wild-type HspB5 to correct F508del-CFTR.

We first showed that a significant increase in HspB5 levels of expression was detected in human nasal epithelial cells (HNEC) cultured from CF patients compared to those from normal individuals [74]. The HNEC cell system is considered as the best in vitro model for testing CFTR modulators [76]. This observation was confirmed in vivo by comparing mice homozygous for the F508del-CFTR mutation to wild-type mice. This study also revealed that HspB5 expression was stimulated, but only in wild-type mice, following intratracheal instillation of LPS from *P. aeruginosa* to promote lung inflammation. We next analyzed the fate of transiently transfected HspB5 in wild-type- and F508del-CFTR expressing CFBE cells that endogenously express HspB1 but not HspB4 or HspB5 [74]. Expression levels of HspB1 and HspB4 were unaffected by HspB5 overexpression. We then observed that, in F508del-CFTR cells, HspB5 was less phosphorylated at the level of serine 19 and serine 59 than in wild-type cells whereas phosphorylation of serine 45 was unchanged. This particular observation, which suggests that in the presence of the F508del-CFTR mutant HspB5 gets dephosphorylated, prompted us to test the transient expression of HspB5 phosphomimetic mutants where serine (S) residues are replaced by alanine (A) to mimic nonphosphorylated residues, or by aspartate (D) to mimic constitutive phosphorylation—towards the transport, function and stability of this mutant polypeptide. In different cell lines, including human bronchial epithelial cell line BEAS-2B, HspB5 was found to increase core-glycosylated F508del-CFTR stability and plasma membrane localization. Moreover, HspB5 increased halide transport in sharp contrast to HspB1 and HspB4 that did the reverse (Figure 4).

Interestingly, one of the HspB5 phosphomimetic mutants (S19D, S45A, S59D identified DAD in the study) appeared to be more effective than the wild-type counterpart to correct F508del-CFTR problems suggesting that specific phosphorylation events modulate HspB5 actions against mutant CFTR [74]. This observation is in perfect agreement with a previous report showing that HspB5 chaperone activity towards multipass transmembrane proteins other than CFTR is finely regulated by phosphorylation [77].

## 5. The Impact of Small HspBs on Existing Therapeutic Corrector/Potentiator Treatments of CFTR

As mentioned above, inhibition of HspB1 expression increases the stability, the intracellular level and therefore the maturation of CFTR mutants. Silencing of HspB1 also increases the canal activity of F508del-CFTR in short-circuit current experiments. The treatment of cells with therapeutic molecules (C4 + C18) currently tested for the treatment of CF diminishes the binding of HspB1 to F508del-CFTR. However, the rescue of the CFTR mutant was not found to be additive or synergistic when the silencing of HspB1 is combined with correctors (C4 + C18) simultaneously [41]. Future studies are needed to establish if silencing of HspB1 could have an additive or synergistic effect with the already FDA approved drugs used to treat Cystic Fibrosis such as Orkambi (VX-770 and VX-809), Symdeco (VX-661 and VX-770) or Trikafta (VX-445, VX-661 and VX-770) or even with the potentiator Ivacaftor (VX-770) alone.

In contrast to the lack of cumulative effects mediated by HspB1 inhibition towards therapeutic agents, we have observed positive properties of HspB5 and moreover of its DAD mutant. These positive outcomes include the stability of core-glycosylated F508del-CFTR, transport at the plasma membrane and anion channel activity. All these positive effects were far more intense in the presence of several therapeutic molecules currently used for the treatment of CF, such as Ivacaftor, Lumacaftor and Orkambi [74]. These data strongly suggest that strategies aiming at improving the expression or delivery of HspB5 should be extensively investigated.

## 6. How to Modulate Intracellular Levels and/or Activities of sHsps or to Exogenously Deliver Them in a CFTR Therapeutic Context

At least three main approaches can be used to modulate the cellular levels or activities of small Hsps: (a) inhibition or stimulation of their expression by specific vectors or compounds, (b) cell delivery of the recombinant protein inserted or not into microparticles (probably the most successful way to enhance sHsps intracellular levels) and (c) modulation of their functions by specific chemicals or peptides [78].

As described above, HspB1 activates the degradation of CFTR bearing NBD1-processing mutations. However, the effects are not uniform and depend on the mutation [41,79,80]. Hence, experiments aimed at decreasing the intracellular level of HspB1 could be an interesting approach to up-regulate the level of CFTR mutants, even if this effect does not appear to be associated with an enhanced protective effect of therapeutic drugs. To that aim, different approaches can be tested, such as decreasing the endogenous level of HspB1 by down-regulating the stability of the protein. Currently, only one compound has been proposed: quercetin (CAS number: 117-39-5). This bioflavonoid is widely distributed in plants and is a well-known natural compound with anti-cancer properties whose mechanism of action has yet to be elucidated [81]. Recent studies have proposed that quercetin could be a regulator of HspB1 stability by inhibiting casein kinase 2 (CK2) which in turn stimulates the degradation of HspB1 by the proteasome [82,83]. It has also been proposed that quercetin regulates the functions of HspB1 by blocking its phosphorylation [84]. However, quercetin has also been identified as a down-regulator of the induction of Hsps which is dependent on the transcription factor HSF1 (Heat Shock Factor 1) [85,86]. This suggests that the impact of this molecule would not be limited to effects on HspB1. In the context of CF where numerous Hsps are involved, the quercetin’s impact appears to be complex to predict. A more specific and promising strategy to decrease the Hsp27 level is the transfection of DNA vectors encoding HspB1 shRNA or siRNA-mediated silencing, such as Apatorsen (OGX-427, CAS number: 1002331-21-6). However, as judged on the immunoblots performed with lung tissues, this type of approach is unable to delete all of the intracellular content of HspB1 [41,87,88]. One possible explanation is that HspB1 has a rather long half-life and therefore a transient destruction of its mRNA has only a limited effect on its cellular level.

Inhibition of HspB1 activity is another approach which could be more effective than RNAi since it can target all HspB1 molecules present in cells. HspB1 mainly acts by interacting with client proteins, which, depending on the pathology, can either be detrimental or beneficial for patients [32,89]. Hence, short peptide aptamers which impair HspB1 interaction with client proteins have been tested. This type of approach could lead to the discovery of potent peptidomimetic drugs. In that regard, we have described peptide aptamers that can either downregulate or stimulate the cytoprotective and anti-apoptotic activities of HspB1 [90]. For example, in mice bearing human head–neck tumors, some aptamers could reduce tumor growth more efficiently than HspB1 depletion through a probable alteration of the pro-cancerous protein interactome associated to this chaperone. Can peptide aptamers be of some interest for cystic fibrosis treatment? The answer could be positive if some aptamers are discovered that can specifically abolish the interaction between HspB1 and CFTR mutants but have no effects towards HspB1 beneficial protein interactome.

Concerning chemicals that target HspB1, only a few are currently tested for their efficiency and specificity, particularly in the cancer field where HspB1 is often considered as a crucial therapeutic target [58]. One can cite the 2’-deoxyuridine derivative RP101 (Brivudine, CAS number: 69304-47-8), an antiviral drug which interacts with HspB1 and inhibits its anti-apoptotic and tumorigenic activities [91]. Based on HspB1-RP101 interaction, a computational drug repositioning approach and use of docking and PLIP (Protein-Ligand Interaction Profiler) interaction patterns led to the discovery of six new HspB1 inhibitors that downregulate HspB1 chaperone activity [92,93]. The most promising molecules are an antipsychotic drug, Chlorpromazine (CAS number: 50-53-3), and a drug used to treat nonimmune subjects with acute malaria attacks, Amodiaquine (CAS number: 86-42-0). Whether these chemicals are of interest for CF is an open question. Anyhow, RP101 is currently tested in pulmonary fibrosis associated with increased HspB1 expression [94]. The outcome of this study will be interesting since RP-101 clinical applications in cancer appear limited due to toxic side effects (Choi et al., 2019). Other inhibitors target its unique cysteine residue, thus impeding its dimerization. This approach is based on the observation that the substitution of this cysteine residue inhibits HspB1 protective activity through inhibition of dimer formation [60,95]. In that respect, chemicals that induce HspB1 potent cysteine-dependent cross-linking are actively searched for. The first compound identified was Zerumbone (CAS number: 471-05-6), a cytotoxic component isolated from Zingiber zerumbet smith [96]. Based on the structure of Zerumbone, several synthetic molecules have been developed and tested. The most promising ones in the CF context being the xanthone derivate SW15 and above all the chromone derivate J2 compound in regard to their lower basal cytotoxicity [94,97,98]. However, caution should be taken with these compounds since their specificity is still not well documented. A similar conclusion can be drawn for different compounds targeting cancer cells where HspB1 has detrimental activity. Usually, these drugs are not highly specific for HspB1 and have side effects. This is well illustrated by Ivermectin (CAS number: 70288-86-7), which potentiates the activity of the anti-androgen receptor and anti-EGFR drugs in prostate and EGFR/HER2-driven tumor models, and also activates GABA-A receptor activity, hence producing neurologic toxicity [99]. Nimesulide (CAS number: 51803-78-2), in addition to inhibiting HspB1 chaperone activity, is also effective towards tubulin polymerization and AR (Androgen Receptor) transcription. However, in this latter case, these cumulative effects appear to be beneficial to counteracting the progression of glioblastoma in mice [100]. Overall, chemical downregulation of HspB1 is beneficial for some cancers. Indeed, aging of the CF population is associated with increased prevalence of comorbidities, such as increased risks of cancer [101,102,103,104]. Hence, it is urgently interesting to address whether cancer HspB1 therapies are compatible, have a positive effect or, at least, are not deleterious with future CF treatments.

What about HspB5? It increases core-glycosylated F508del-CFTR stability, plasma membrane localization and halide transport. Hence, an obvious approach is to positively increase its intracellular level. Another interesting effect of HspB5 is its role in inflammation, since in CF, inflammation plays a critical role in disease progression. Indeed, the delivery of exogenous HspB5 is already an active field of research consequently of its intrinsic anti-inflammatory activity. To this end, a considerable promising approach is the direct delivery of this protein through its association to porous Poly (lactic-co-glycolic acid) (PGLA) microparticles [105]. This approach was assessed in a mouse model of chronic obstructive pulmonary disease (COPD). Intratracheal administration of PGLA-HspB5 microparticles significantly suppresses lung infiltration by lymphocytes and neutrophils, in contrast to the inefficient administration of free soluble HspB5 [105]. Moreover, a human randomized Multiple Sclerosis controlled phase IIa trial shows that exogenous PGLA-HspB5 (DC-TAB) administration is safe, well-tolerated and beneficial for patients [106].

In the CF context, a precise phosphorylated form of HspB5 is the most potent, which indicates that its activity needs to be modified more than its intracellular level. As described with HspB1, peptide aptamers could be considered since no chemical has yet been described to specifically modulate HspB5 activity. In that regard, we have already described a peptide aptamer that targets HspB5 and modulates its activity [106], hence demonstrating the feasibility of the approach.

## 7. Conclusions

In view of the data presented in this review, one can easily conclude that, in the context of CF, sHsps are fascinating therapeutic targets. As already observed in other diseases, these proteins are unpredictable since they will have to be either stimulated or inhibited to provide a positive outcome. Therefore, there is clearly a lot more investigations to perform. In the near future, this field of research will rapidly expand since other additional members of the sHsps human family are constitutively expressed in lungs. However, the task may be intense since sHsps can form complex oligomers that probably modulate their activities. Hence, we strongly believe that future research in these domains may bring new conditions or compounds that could enhance the biological understanding of CFTR processing and have a positive clinical outcome for CF patients.

## Data Availability

Not applicable.

## References

[1] Riordan J.R., Rommens J.M., Kerem B., Alon N., Rozmahel R., Grzelczak Z., Zielenski J., Lok S., Plavsic N., Chou J.L. (1989). Identification of the Cystic Fibrosis Gene: Cloning and Characterization of Complementary DNA. Science.

[2] Hyde S.C., Emsley P., Hartshorn M.J., Mimmack M.M., Gileadi U., Pearce S.R., Gallagher M.P., Gill D.R., Hubbard R.E., Higgins C.F. (1990). Structural Model of ATP-Binding Proteins Associated with Cystic Fibrosis, Multidrug Resistance and Bacterial Transport. Nature.

[3] Zhang Z., Liu F., Chen J. (2017). Conformational Changes of CFTR upon Phosphorylation and ATP Binding. Cell.

[4] Hwang T.-C., Sheppard D.N. (2009). Gating of the CFTR Cl- Channel by ATP-Driven Nucleotide-Binding Domain Dimerisation. J. Physiol..

[5] Crawford I., Maloney P.C., Zeitlin P.L., Guggino W.B., Hyde S.C., Turley H., Gatter K.C., Harris A., Higgins C.F. (1991). Immunocytochemical Localization of the Cystic Fibrosis Gene Product CFTR. Proc. Natl. Acad. Sci. USA.

[6] Frizzell R.A., Hanrahan J.W. (2012). Physiology of Epithelial Chloride and Fluid Secretion. Cold Spring Harb. Perspect. Med..

[7] Mall M., Bleich M., Greger R., Schreiber R., Kunzelmann K. (1998). The Amiloride-Inhibitable Na^+^ Conductance Is Reduced by the Cystic Fibrosis Transmembrane Conductance Regulator in Normal but Not in Cystic Fibrosis Airways. J. Clin. Investig..

[8] Schwiebert E.M., Benos D.J., Egan M.E., Stutts M.J., Guggino W.B. (1999). CFTR Is a Conductance Regulator as Well as a Chloride Channel. Physiol. Rev..

[9] Wei L., Vankeerberghen A., Cuppens H., Cassiman J.J., Droogmans G., Nilius B. (2001). The C-Terminal Part of the R-Domain, but Not the PDZ Binding Motif, of CFTR Is Involved in Interaction with Ca^2+^-Activated Cl^−^ Channels. Pflugers Arch..

[10] Vij N., Mazur S., Zeitlin P.L. (2009). CFTR Is a Negative Regulator of NFkappaB Mediated Innate Immune Response. PLoS ONE.

[11] Carveth K., Buck T., Anthony V., Skach W.R. (2002). Cooperativity and Flexibility of Cystic Fibrosis Transmembrane Conductance Regulator Transmembrane Segments Participate in Membrane Localization of a Charged Residue. J. Biol. Chem..

[12] Pitonzo D., Yang Z., Matsumura Y., Johnson A.E., Skach W.R. (2009). Sequence-Specific Retention and Regulated Integration of a Nascent Membrane Protein by the Endoplasmic Reticulum Sec61 Translocon. Mol. Biol. Cell.

[13] Kleizen B., van Vlijmen T., de Jonge H.R., Braakman I. (2005). Folding of CFTR Is Predominantly Cotranslational. Mol. Cell.

[14] Farinha C.M., Canato S. (2017). From the Endoplasmic Reticulum to the Plasma Membrane: Mechanisms of CFTR Folding and Trafficking. Cell. Mol. Life Sci..

[15] Meacham G.C., Lu Z., King S., Sorscher E., Tousson A., Cyr D.M. (1999). The Hdj-2/Hsc70 Chaperone Pair Facilitates Early Steps in CFTR Biogenesis. EMBO J..

[16] Wang X., Venable J., LaPointe P., Hutt D.M., Koulov A.V., Coppinger J., Gurkan C., Kellner W., Matteson J., Plutner H. (2006). Hsp90 Cochaperone Aha1 Downregulation Rescues Misfolding of CFTR in Cystic Fibrosis. Cell.

[17] Skach W.R. (2006). CFTR: New Members Join the Fold. Cell.

[18] Younger J.M., Chen L., Ren H.-Y., Rosser M.F.N., Turnbull E.L., Fan C.-Y., Patterson C., Cyr D.M. (2006). Sequential Quality-Control Checkpoints Triage Misfolded Cystic Fibrosis Transmembrane Conductance Regulator. Cell.

[19] Wang X., Matteson J., An Y., Moyer B., Yoo J.-S., Bannykh S., Wilson I.A., Riordan J.R., Balch W.E. (2004). COPII-Dependent Export of Cystic Fibrosis Transmembrane Conductance Regulator from the ER Uses a Di-Acidic Exit Code. J. Cell Biol..

[20] Cheng S.H., Gregory R.J., Marshall J., Paul S., Souza D.W., White G.A., O’Riordan C.R., Smith A.E. (1990). Defective Intracellular Transport and Processing of CFTR Is the Molecular Basis of Most Cystic Fibrosis. Cell.

[21] Riordan J.R. (2008). CFTR Function and Prospects for Therapy. Annu. Rev. Biochem..

[22] Johnston J.A., Ward C.L., Kopito R.R. (1998). Aggresomes: A Cellular Response to Misfolded Proteins. J. Cell Biol..

[23] Chua C.E.L., Lim Y.S., Lee M.G., Tang B.L. (2012). Non-Classical Membrane Trafficking Processes Galore. J. Cell. Physiol..

[24] Gee H.Y., Noh S.H., Tang B.L., Kim K.H., Lee M.G. (2011). Rescue of ΔF508-CFTR Trafficking via a GRASP-Dependent Unconventional Secretion Pathway. Cell.

[25] Yoo J.-S., Moyer B.D., Bannykh S., Yoo H.-M., Riordan J.R., Balch W.E. (2002). Non-Conventional Trafficking of the Cystic Fibrosis Transmembrane Conductance Regulator through the Early Secretory Pathway. J. Biol. Chem..

[26] Amaral M.D., Hutt D.M., Tomati V., Botelho H.M., Pedemonte N. (2020). CFTR Processing, Trafficking and Interactions. J. Cyst. Fibros. Off. J. Eur. Cyst. Fibros. Soc..

[27] Ingolia T.D., Craig E.A. (1982). Four Small Drosophila Heat Shock Proteins Are Related to Each Other and to Mammalian α-Crystallin. Proc. Natl. Acad. Sci. USA.

[28] Kappé G., Franck E., Verschuure P., Boelens W.C., Leunissen J.A.M., de Jong W.W. (2003). The Human Genome Encodes 10 A-Crystallin-Related Small Heat Shock Proteins: HspB1-10. Cell Stress Chaperones.

[29] Taylor R.P., Benjamin I.J. (2005). Small Heat Shock Proteins: A New Classification Scheme in Mammals. J. Mol. Cell. Cardiol..

[30] Kampinga H.H., Hageman J., Vos M.J., Kubota H., Tanguay R.M., Bruford E.A., Cheetham M.E., Chen B., Hightower L.E. (2009). Guidelines for the Nomenclature of the Human Heat Shock Proteins. Cell Stress Chaperones.

[31] Arrigo A.-P., Gibert B. (2013). Protein Interactomes of Three Stress Inducible Small Heat Shock Proteins: HspB1, HspB5 and HspB8. Int. J. Hyperth..

[32] Arrigo A.-P. (2013). Human Small Heat Shock Proteins: Protein Interactomes of Homo- and Hetero-Oligomeric Complexes: An Update. FEBS Lett..

[33] Qu B.H., Strickland E., Thomas P.J. (1997). Cystic Fibrosis: A Disease of Altered Protein Folding. J. Bioenerg. Biomembr..

[34] Loo M.A., Jensen T.J., Cui L., Hou Y., Chang X.B., Riordan J.R. (1998). Perturbation of Hsp90 Interaction with Nascent CFTR Prevents Its Maturation and Accelerates Its Degradation by the Proteasome. EMBO J..

[35] Rubenstein R.C., Zeitlin P.L. (2000). Sodium 4-Phenylbutyrate Downregulates Hsc70: Implications for Intracellular Trafficking of DeltaF508-CFTR. Am. J. Physiol. Cell Physiol..

[36] Choo-Kang L.R., Zeitlin P.L. (2001). Induction of HSP70 Promotes ΔF508 CFTR Trafficking. Am. J. Physiol. Lung Cell. Mol. Physiol..

[37] Farinha C.M., Nogueira P., Mendes F., Penque D., Amaral M.D. (2002). The Human DnaJ Homologue (Hdj)-1/Heat-Shock Protein (Hsp) 40 Co-Chaperone Is Required for the In Vivo Stabilization of the Cystic Fibrosis Transmembrane Conductance Regulator by Hsp70. Biochem. J..

[38] Alberti S., Böhse K., Arndt V., Schmitz A., Höhfeld J. (2004). The Cochaperone HspBP1 Inhibits the CHIP Ubiquitin Ligase and Stimulates the Maturation of the Cystic Fibrosis Transmembrane Conductance Regulator. Mol. Biol. Cell.

[39] Youker R.T., Walsh P., Beilharz T., Lithgow T., Brodsky J.L. (2004). Distinct Roles for the Hsp40 and Hsp90 Molecular Chaperones during Cystic Fibrosis Transmembrane Conductance Regulator Degradation in Yeast. Mol. Biol. Cell.

[40] Zhang H., Schmidt B.Z., Sun F., Condliffe S.B., Butterworth M.B., Youker R.T., Brodsky J.L., Aridor M., Frizzell R.A. (2006). Cysteine String Protein Monitors Late Steps in Cystic Fibrosis Transmembrane Conductance Regulator Biogenesis. J. Biol. Chem..

[41] Lopes-Pacheco M., Boinot C., Sabirzhanova I., Morales M.M., Guggino W.B., Cebotaru L. (2015). Combination of Correctors Rescue ΔF508-CFTR by Reducing Its Association with Hsp40 and Hsp27. J. Biol. Chem..

[42] Rubenstein R.C., Zeitlin P.L. (1998). A Pilot Clinical Trial of Oral Sodium 4-Phenylbutyrate (Buphenyl) in DeltaF508-Homozygous Cystic Fibrosis Patients: Partial Restoration of Nasal Epithelial CFTR Function. Am. J. Respir. Crit. Care Med..

[43] Rubenstein R.C., Egan M.E., Zeitlin P.L. (1997). In Vitro Pharmacologic Restoration of CFTR-Mediated Chloride Transport with Sodium 4-Phenylbutyrate in Cystic Fibrosis Epithelial Cells Containing Delta F508-CFTR. J. Clin. Investig..

[44] Suaud L., Miller K., Panichelli A.E., Randell R.L., Marando C.M., Rubenstein R.C. (2011). 4-Phenylbutyrate Stimulates Hsp70 Expression through the Elp2 Component of Elongator and STAT-3 in Cystic Fibrosis Epithelial Cells. J. Biol. Chem..

[45] Colunga Biancatelli R.M.L., Solopov P., Gregory B., Catravas J.D. (2020). HSP90 Inhibition and Modulation of the Proteome: Therapeutical Implications for Idiopathic Pulmonary Fibrosis (IPF). Int. J. Mol. Sci..

[46] Horwitz J., Bova M.P., Ding L.L., Haley D.A., Stewart P.L. (1999). Lens A-Crystallin: Function and Structure. Eye Lond. Engl..

[47] Liu J., Luo Z., Zhang L., Wang L., Nie Q., Wang Z.-F., Huang Z., Hu X., Gong L., Arrigo A.-P. (2016). The Small Heat Shock Protein AA-Crystallin Negatively Regulates Pancreatic Tumorigenesis. Oncotarget.

[48] Hansen L., Yao W., Eiberg H., Kjaer K.W., Baggesen K., Hejtmancik J.F., Rosenberg T. (2007). Genetic Heterogeneity in Microcornea-Cataract: Five Novel Mutations in CRYAA, CRYGD, and GJA8. Investig. Ophthalmol. Vis. Sci..

[49] Devi R.R., Yao W., Vijayalakshmi P., Sergeev Y.V., Sundaresan P., Hejtmancik J.F. (2008). Crystallin Gene Mutations in Indian Families with Inherited Pediatric Cataract. Mol. Vis..

[50] Graw J., Klopp N., Illig T., Preising M.N., Lorenz B. (2006). Congenital Cataract and Macular Hypoplasia in Humans Associated with a de Novo Mutation in CRYAA and Compound Heterozygous Mutations in P. Graefes Arch. Clin. Exp. Ophthalmol..

[51] Andley U.P. (2009). Effects of A-Crystallin on Lens Cell Function and Cataract Pathology. Curr. Mol. Med..

[52] Ahner A., Nakatsukasa K., Zhang H., Frizzell R.A., Brodsky J.L. (2007). Small Heat-Shock Proteins Select DeltaF508-CFTR for Endoplasmic Reticulum-Associated Degradation. Mol. Biol. Cell.

[53] Kashlan O.B., Mueller G.M., Qamar M.Z., Poland P.A., Ahner A., Rubenstein R.C., Hughey R.P., Brodsky J.L., Kleyman T.R. (2007). Small Heat Shock Protein AA-Crystallin Regulates Epithelial Sodium Channel Expression. J. Biol. Chem..

[54] Arrigo A.-P., Simon S., Gibert B., Kretz-Remy C., Nivon M., Czekalla A., Guillet D., Moulin M., Diaz-Latoud C., Vicart P. (2007). Hsp27 (HspB1) and αB-Crystallin (HspB5) as Therapeutic Targets. FEBS Lett..

[55] Ciocca D.R., Arrigo A.P., Calderwood S.K. (2013). Heat Shock Proteins and Heat Shock Factor 1 in Carcinogenesis and Tumor Development: An Update. Arch. Toxicol..

[56] Shiota M., Bishop J.L., Nip K.M., Zardan A., Takeuchi A., Cordonnier T., Beraldi E., Bazov J., Fazli L., Chi K. (2013). Hsp27 Regulates Epithelial Mesenchymal Transition, Metastasis, and Circulating Tumor Cells in Prostate Cancer. Cancer Res..

[57] Arrigo A.-P., Gibert B. (2014). HspB1, HspB5 and HspB4 in Human Cancers: Potent Oncogenic Role of Some of Their Client Proteins. Cancers.

[58] Arrigo A.P., Suhan J.P., Welch W.J. (1988). Dynamic Changes in the Structure and Intracellular Locale of the Mammalian Low-Molecular-Weight Heat Shock Protein. Mol. Cell. Biol..

[59] Arrigo A.-P. (2005). Heat shock proteins as molecular chaperones. Med. Sci..

[60] Arrigo A.-P. (2007). The Cellular “Networking” of Mammalian Hsp27 and Its Functions in the Control of Protein Folding, Redox State and Apoptosis. Adv. Exp. Med. Biol..

[61] Paul C., Simon S., Gibert B., Virot S., Manero F., Arrigo A.-P. (2010). Dynamic Processes That Reflect Anti-Apoptotic Strategies Set up by HspB1 (Hsp27). Exp. Cell Res..

[62] Arrigo A.-P. (2017). Mammalian HspB1 (Hsp27) Is a Molecular Sensor Linked to the Physiology and Environment of the Cell. Cell Stress Chaperones.

[63] Gibert B., Eckel B., Fasquelle L., Moulin M., Bouhallier F., Gonin V., Mellier G., Simon S., Kretz-Remy C., Arrigo A.-P. (2012). Knock down of Heat Shock Protein 27 (HspB1) Induces Degradation of Several Putative Client Proteins. PLoS ONE.

[64] Arrigo A.-P., Gibert B. (2012). HspB1 Dynamic Phospho-Oligomeric Structure Dependent Interactome as Cancer Therapeutic Target. Curr. Mol. Med..

[65] Muranova L.K., Sudnitsyna M.V., Strelkov S.V., Gusev N.B. (2020). Mutations in HspB1 and Hereditary Neuropathies. Cell Stress Chaperones.

[66] Benndorf R., Martin J.L., Kosakovsky Pond S.L., Wertheim J.O. (2014). Neuropathy- and Myopathy-Associated Mutations in Human Small Heat Shock Proteins: Characteristics and Evolutionary History of the Mutation Sites. Mutat. Res..

[67] Roxo-Rosa M., da Costa G., Luider T.M., Scholte B.J., Coelho A.V., Amaral M.D., Penque D. (2006). Proteomic Analysis of Nasal Cells from Cystic Fibrosis Patients and Non-Cystic Fibrosis Control Individuals: Search for Novel Biomarkers of Cystic Fibrosis Lung Disease. Proteomics.

[68] Ahner A., Gong X., Schmidt B.Z., Peters K.W., Rabeh W.M., Thibodeau P.H., Lukacs G.L., Frizzell R.A. (2013). Small Heat Shock Proteins Target Mutant Cystic Fibrosis Transmembrane Conductance Regulator for Degradation via a Small Ubiquitin-like Modifier-Dependent Pathway. Mol. Biol. Cell.

[69] Ahner A., Gong X., Frizzell R.A. (2013). Cystic Fibrosis Transmembrane Conductance Regulator Degradation: Cross-Talk between the Ubiquitylation and SUMOylation Pathways. FEBS J..

[70] Ahner A., Gong X., Frizzell R.A. (2016). Divergent Signaling via SUMO Modification: Potential for CFTR Modulation. Am. J. Physiol. Cell Physiol..

[71] Gong X., Ahner A., Roldan A., Lukacs G.L., Thibodeau P.H., Frizzell R.A. (2016). Non-Native Conformers of Cystic Fibrosis Transmembrane Conductance Regulator NBD1 Are Recognized by Hsp27 and Conjugated to SUMO-2 for Degradation. J. Biol. Chem..

[72] Gong X., Liao Y., Ahner A., Larsen M.B., Wang X., Bertrand C.A., Frizzell R.A. (2019). Different SUMO Paralogues Determine the Fate of Wild-Type and Mutant CFTRs: Biogenesis versus Degradation. Mol. Biol. Cell.

[73] Degrugillier F., Aissat A., Prulière-Escabasse V., Bizard L., Simonneau B., Decrouy X., Jiang C., Rotin D., Fanen P., Simon S. (2020). Phosphorylation of the Chaperone-Like HspB5 Rescues Trafficking and Function of F508del-CFTR. Int. J. Mol. Sci..

[74] D’Agostino M., Lemma V., Chesi G., Stornaiuolo M., Cannata Serio M., D’Ambrosio C., Scaloni A., Polishchuk R., Bonatti S. (2013). The Cytosolic Chaperone α-Crystallin B Rescues Folding and Compartmentalization of Misfolded Multispan Transmembrane Proteins. J. Cell Sci..

[75] Pranke I.M., Hatton A., Simonin J., Jais J.P., Le Pimpec-Barthes F., Carsin A., Bonnette P., Fayon M., Stremler-Le Bel N., Grenet D. (2017). Correction of CFTR Function in Nasal Epithelial Cells from Cystic Fibrosis Patients Predicts Improvement of Respiratory Function by CFTR Modulators. Sci. Rep..

[76] Ciano M., Allocca S., Ciardulli M.C., Della Volpe L., Bonatti S., D’Agostino M. (2016). Differential Phosphorylation-Based Regulation of AB-Crystallin Chaperone Activity for Multipass Transmembrane Proteins. Biochem. Biophys. Res. Commun..

[77] Choi S.-K., Kam H., Kim K.-Y., Park S.I., Lee Y.-S. (2019). Targeting Heat Shock Protein 27 in Cancer: A Druggable Target for Cancer Treatment?. Cancers.

[78] Lopes-Pacheco M., Sabirzhanova I., Rapino D., Morales M.M., Guggino W.B., Cebotaru L. (2016). Correctors Rescue CFTR Mutations in Nucleotide-Binding Domain 1 (NBD1) by Modulating Proteostasis. Chembiochem Eur. J. Chem. Biol..

[79] Lopes-Pacheco M., Boinot C., Sabirzhanova I., Rapino D., Cebotaru L. (2017). Combination of Correctors Rescues CFTR Transmembrane-Domain Mutants by Mitigating Their Interactions with Proteostasis. Cell. Physiol. Biochem. Int. J. Exp. Cell. Physiol. Biochem. Pharmacol..

[80] Murakami A., Ashida H., Terao J. (2008). Multitargeted Cancer Prevention by Quercetin. Cancer Lett..

[81] Borgo C., Vilardell J., Bosello-Travain V., Pinna L.A., Venerando A., Salvi M. (2018). Dependence of HSP27 Cellular Level on Protein Kinase CK2 Discloses Novel Therapeutic Strategies. Biochim. Biophys. Acta Gen. Subj..

[82] Russo M., Milito A., Spagnuolo C., Carbone V., Rosén A., Minasi P., Lauria F., Russo G.L. (2017). CK2 and PI3K Are Direct Molecular Targets of Quercetin in Chronic Lymphocytic Leukaemia. Oncotarget.

[83] Sang D.-P., Li R.-J., Lan Q. (2014). Quercetin Sensitizes Human Glioblastoma Cells to Temozolomide in Vitro via Inhibition of Hsp27. Acta Pharmacol. Sin..

[84] Hosokawa N., Hirayoshi K., Kudo H., Takechi H., Aoike A., Kawai K., Nagata K. (1992). Inhibition of the Activation of Heat Shock Factor in Vivo and in Vitro by Flavonoids. Mol. Cell. Biol..

[85] Nagai N., Nakai A., Nagata K. (1995). Quercetin Suppresses Heat Shock Response by down Regulation of HSF1. Biochem. Biophys. Res. Commun..

[86] Lelj-Garolla B., Kumano M., Beraldi E., Nappi L., Rocchi P., Ionescu D.N., Fazli L., Zoubeidi A., Gleave M.E. (2015). Hsp27 Inhibition with OGX-427 Sensitizes Non-Small Cell Lung Cancer Cells to Erlotinib and Chemotherapy. Mol. Cancer Ther..

[87] Spigel D.R., Shipley D.L., Waterhouse D.M., Jones S.F., Ward P.J., Shih K.C., Hemphill B., McCleod M., Whorf R.C., Page R.D. (2019). A Randomized, Double-Blinded, Phase II Trial of Carboplatin and Pemetrexed with or without Apatorsen (OGX-427) in Patients with Previously Untreated Stage IV Non-Squamous-Non-Small-Cell Lung Cancer: The SPRUCE Trial. Oncologist.

[88] Simon S., Arrigo A.-P., Simon S., Arrigo A.P. (2010). Beneficial and Deleterious, the Dual Role of Small Stress Proteins in Human Diseases: Implications for Therapeutic Strategies.

[89] Gibert B., Hadchity E., Czekalla A., Aloy M.-T., Colas P., Rodriguez-Lafrasse C., Arrigo A.-P., Diaz-Latoud C. (2011). Inhibition of Heat Shock Protein 27 (HspB1) Tumorigenic Functions by Peptide Aptamers. Oncogene.

[90] Heinrich J.-C., Tuukkanen A., Schroeder M., Fahrig T., Fahrig R. (2011). RP101 (Brivudine) Binds to Heat Shock Protein HSP27 (HSPB1) and Enhances Survival in Animals and Pancreatic Cancer Patients. J. Cancer Res. Clin. Oncol..

[91] Heinrich J.C., Donakonda S., Haupt V.J., Lennig P., Zhang Y., Schroeder M. (2016). New HSP27 Inhibitors Efficiently Suppress Drug Resistance Development in Cancer Cells. Oncotarget.

[92] Salentin S., Adasme M.F., Heinrich J.C., Haupt V.J., Daminelli S., Zhang Y., Schroeder M. (2017). From Malaria to Cancer: Computational Drug Repositioning of Amodiaquine Using PLIP Interaction Patterns. Sci. Rep..

[93] Kim J.H., Jung Y.J., Choi B., Lee N.L., Lee H.J., Kwak S.Y., Kwon Y., Na Y., Lee Y.-S. (2016). Overcoming HSP27-Mediated Resistance by Altered Dimerization of HSP27 Using Small Molecules. Oncotarget.

[94] Diaz-Latoud C., Buache E., Javouhey E., Arrigo A.-P. (2005). Substitution of the Unique Cysteine Residue of Murine Hsp25 Interferes with the Protective Activity of This Stress Protein through Inhibition of Dimer Formation. Antioxid. Redox Signal..

[95] Choi S.-H., Lee Y.-J., Seo W.D., Lee H.-J., Nam J.-W., Lee Y.J., Kim J., Seo E.-K., Lee Y.-S. (2011). Altered Cross-Linking of HSP27 by Zerumbone as a Novel Strategy for Overcoming HSP27-Mediated Radioresistance. Int. J. Radiat. Oncol. Biol. Phys..

[96] Choi B., Choi S.-K., Park Y.N., Kwak S.-Y., Lee H.J., Kwon Y., Na Y., Lee Y.-S. (2017). Sensitization of Lung Cancer Cells by Altered Dimerization of HSP27. Oncotarget.

[97] Hwang S.-Y., Kwak S.Y., Kwon Y., Lee Y.-S., Na Y. (2017). Synthesis and Biological Effect of Chrom-4-One Derivatives as Functional Inhibitors of Heat Shock Protein 27. Eur. J. Med. Chem..

[98] Nappi L., Aguda A.H., Nakouzi N.A., Lelj-Garolla B., Beraldi E., Lallous N., Thi M., Moore S., Fazli L., Battsogt D. (2020). Ivermectin Inhibits HSP27 and Potentiates Efficacy of Oncogene Targeting in Tumor Models. J. Clin. Investig..

[99] Yi X., Zhong B., Smith K.M., Geldenhuys W.J., Feng Y., Pink J.J., Dowlati A., Xu Y., Zhou A., Su B. (2012). Identification of a Class of Novel Tubulin Inhibitors. J. Med. Chem..

[100] Bell S.C., Mall M.A., Gutierrez H., Macek M., Madge S., Davies J.C., Burgel P.-R., Tullis E., Castaños C., Castellani C. (2020). The Future of Cystic Fibrosis Care: A Global Perspective. Lancet Respir. Med..

[101] Regard L., Martin C., Chassagnon G., Burgel P.-R. (2019). Acute and Chronic Non-Pulmonary Complications in Adults with Cystic Fibrosis. Expert Rev. Respir. Med..

[102] Regard L., Lafoeste H., Martin C., Chassagnon G., Burgel P.-R. (2018). Ageing with cystic fibrosis: Classical and emerging comorbidities in adults with cystic fibrosis. Rev. Pneumol. Clin..

[103] Yamada A., Komaki Y., Komaki F., Micic D., Zullow S., Sakuraba A. (2018). Risk of Gastrointestinal Cancers in Patients with Cystic Fibrosis: A Systematic Review and Meta-Analysis. Lancet Oncol..

[104] van Noort J.M., Bsibsi M., Nacken P.J., Gerritsen W.H., Amor S., Holtman I.R., Boddeke E., van Ark I., Leusink-Muis T., Folkerts G. (2013). Activation of an Immune-Regulatory Macrophage Response and Inhibition of Lung Inflammation in a Mouse Model of COPD Using Heat-Shock Protein αB-Crystallin-Loaded PLGA Microparticles. Biomaterials.

[105] van Noort J.M., Bsibsi M., Nacken P.J., Verbeek R., Venneker E.H.G. (2015). Therapeutic Intervention in Multiple Sclerosis with A B-Crystallin: A Randomized Controlled Phase IIa Trial. PLoS ONE.

[106] Gibert B., Simon S., Dimitrova V., Diaz-Latoud C., Arrigo A.-P. (2013). Peptide Aptamers: Tools to Negatively or Positively Modulate HSPB1(27) Function. Philos. Trans. R. Soc. Lond. B. Biol. Sci..

